# Cutaneous Larva Migrans with Löeffler’s Syndrome

**DOI:** 10.4269/ajtmh.18-0406

**Published:** 2019-03

**Authors:** Ya-Li Gao, Ze-Hu Liu

**Affiliations:** Department of Dermatology, Affiliated Third Hospital of Hangzhou, Anhui Medical University, Hangzhou, China

**Figure 1.**
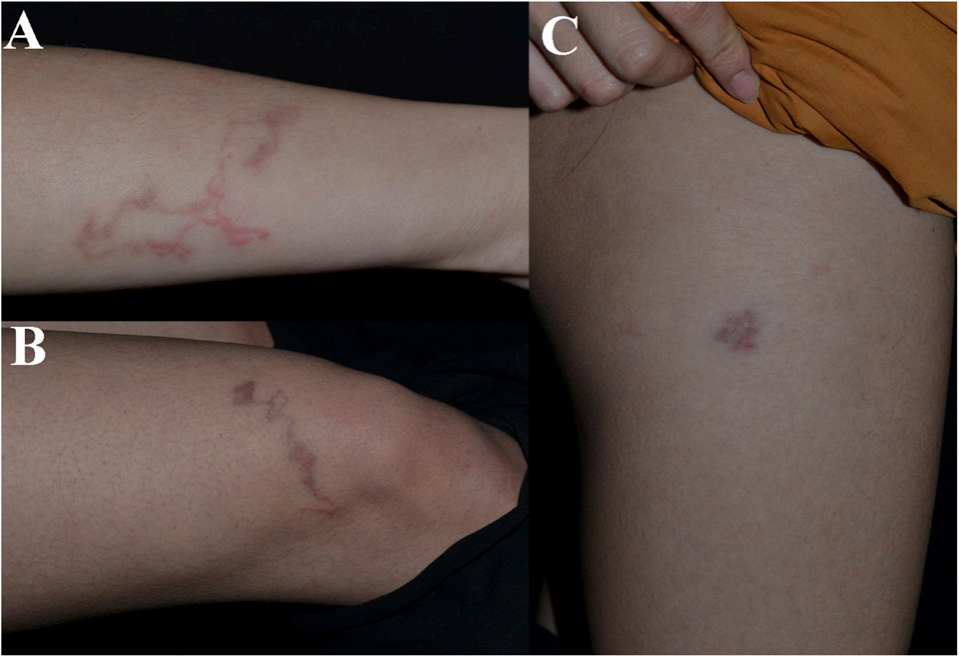
Serpiginous erythematous track on the right upper extremity (**A**) and lower extremity (**B**, **C**). This figure appears in color at www.ajtmh.org.

A 26-year-old woman presented with itching serpiginous, slightly elevated, erythematous tracks on her right upper (Figure 1A) and lower extremity (Figure 1B, C) over a period of 2 weeks. She also referred nonproductive cough and occasional breathlessness for 1 week. She did not recall a history of an insect bite, yet she had traveled to Sabah, Malaysia, a month ago. She confessed that she had buried her body in the sand.

**Figure 2.**
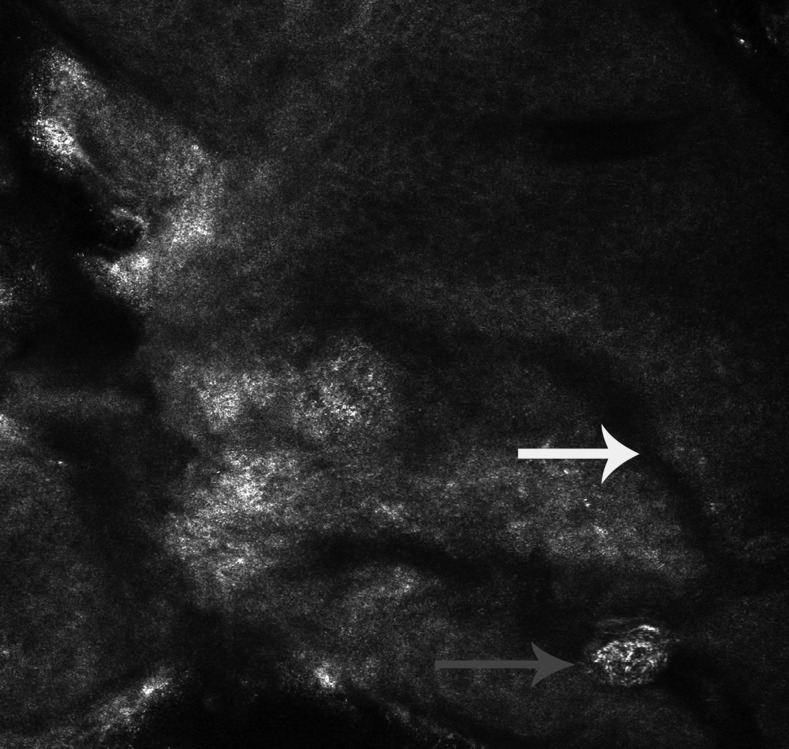
Reflectance confocal microscopy showed burrow (white arrow) and a highly refractile larva (red arrow) in the epidermis.

**Figure 3.**
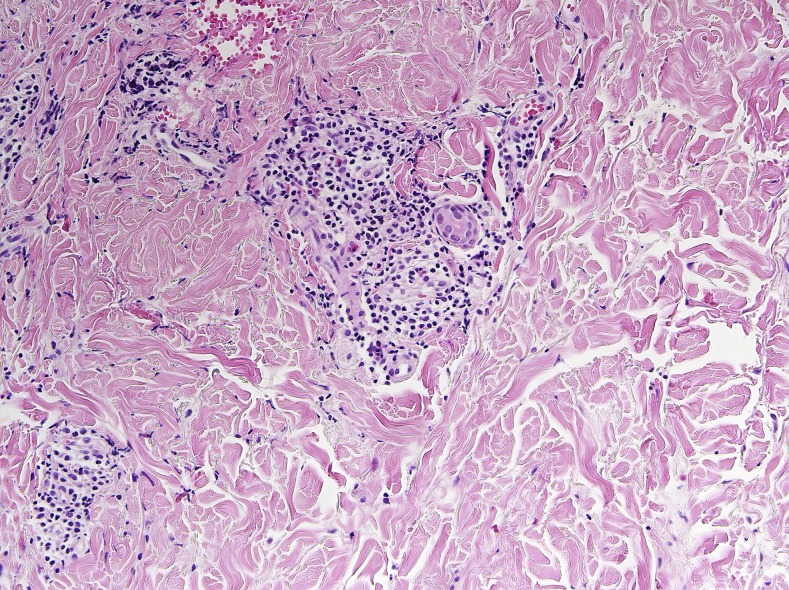
Histopathology showed only prominent infiltration of eosinophils around the vessels in the dermis. This figure appears in color at www.ajtmh.org.

**Figure 4.**
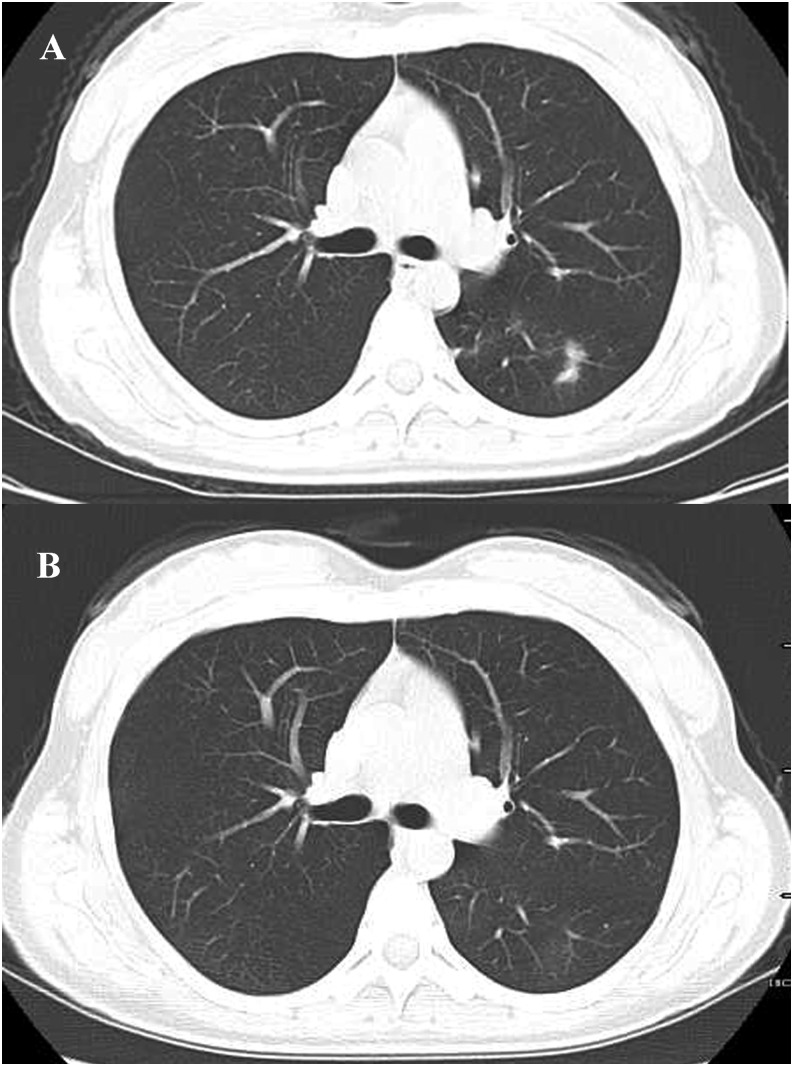
CT scan showed ill-defined reticulonodular infiltrates in both lungs (**A**), and disappeared 10 days after treatment (**B**).

Reflectance confocal microscopy showed burrow and a highly refractile oval larva in the epidermis (Figure 2). Laboratory studies showed mild eosinophilia. Histopathology showed only prominent infiltration of eosinophils around the vessels in the dermis without a hookworm larva (Figure 3). The computed tomography (CT) scan showed ill-defined reticulonodular infiltrates in both lungs (Figure 4A). Thus, she was diagnosed with cutaneous larva migrans (CLM) with Löeffler’s syndrome. The patient was treated with oral albendazole 400 mg for seven consecutive days. The lesions stopped creeping and itch was relieved within 24 hours. Peripheral eosinophilia normalized and breathlessness resolved 7 days later. In the follow-up CT scan, the reticulonodular infiltrates in lungs had disappeared (Figure 4B).

Cutaneous larva migrans is one of the most frequent travel-associated skin diseases on beach destinations in the tropics or subtropics. This location mirrors the geographic distribution of *Ancylostoma braziliense*. The most frequent anatomic locations of CLM lesions are the lower extremities (73.4%).^1^ The larvae migrate aimlessly within the epidermis at a rate of a couple of millimeters to 2 cm a day, for it lacks the enzymes necessary to penetrate and survive in the deeper dermis.^2^

Cutaneous larva migrans is usually confined to the skin, where it causes creeping eruption and rarely results in severe infestations. However, it can also cause pulmonary and intestinal symptoms,^3^ leading to eosinophilic enteritis or Löeffler’s syndrome. This is characterized by the pulmonary infiltrates and eosinophilia, and is considered to be an immunologic reaction to the larvae, regardless of whether the larvae remain in the skin or have migrated to the lungs.^4,5^ Differential diagnosis includes other insect bite reactions, tinea corporis, erythema chronicum migrans, and contact dermatitis. Oral ivermectin or/and albendazole are effective treatment options for this self-limiting disease.

Our case highlights that it is important for physicians to consider a CLM infection in patients with cough and breathlessness associated with itching creeping eruptions in skin, especially if there is a history of recent travel to the tropics and contact with sand.
